# Supplementary figures and images for: Distinct DNA-binding surfaces in the ATPase and linker domains of MutLγ determine its substrate specificities and exert separable functions in meiotic recombination and mismatch repair
Source: PLoS Genet. 2017 May 15;13(5):e1006722. doi: 10.1371/journal.pgen.1006722 (PMC5448812; doi:10.1371/journal.pgen.1006722)

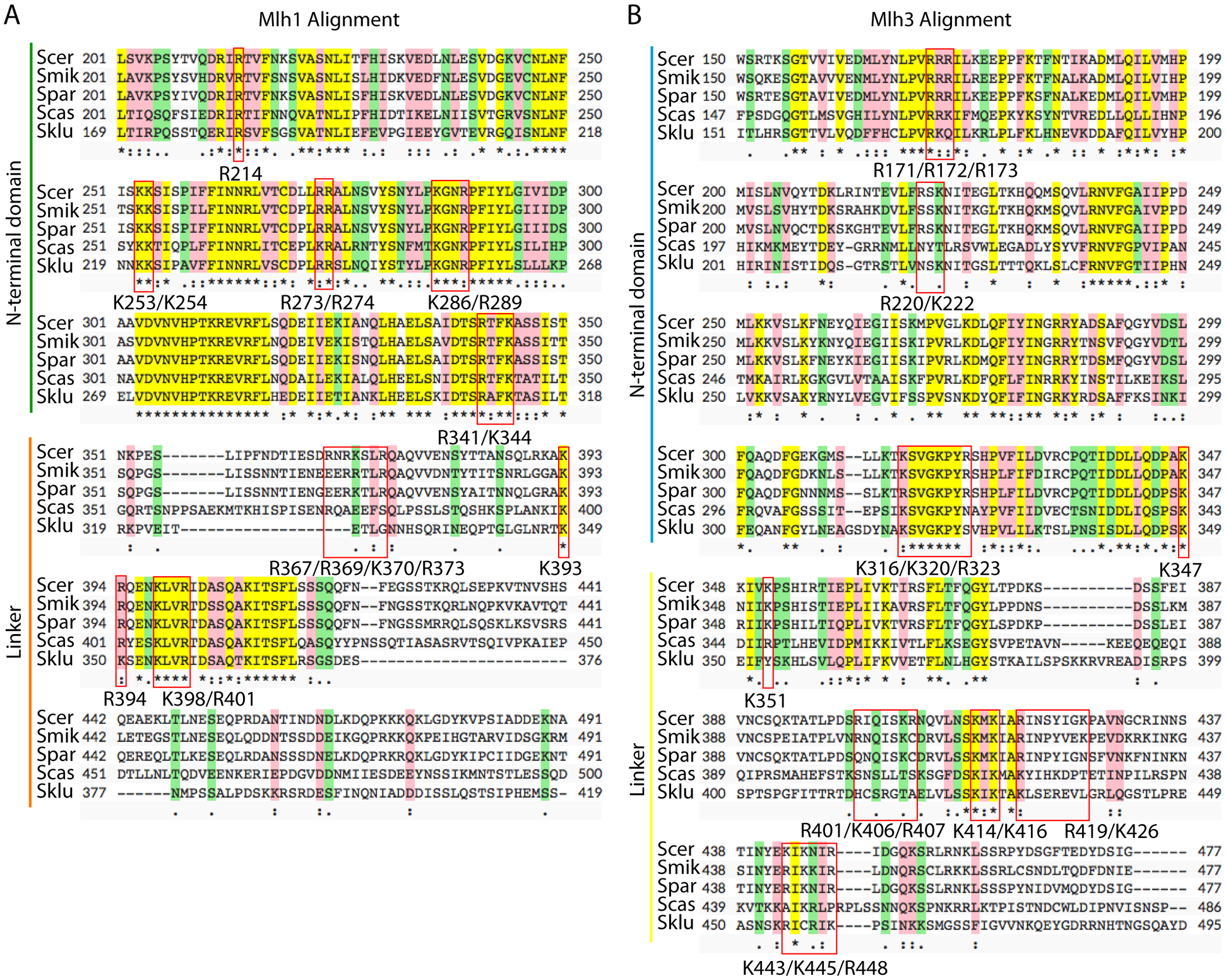

Supplement: S1 Fig — Alignments were generated using the Saccharomyces Genome Databank. Scer: S. cerevisiae; Smik, S. mikatae; Spar, S. paradoxus; Scas, S. castellii; Sklu, S. kluyveri. Yellow indicates conserved residues; pink indicates strong similarity; green indicates weak similarity. Red boxes highlight regions of Mlh1 (A) and Mlh3 (B) that contain lysine and arginine residues that were mutated for functional analyses. (TIF) [file pgen.1006722.s001.tif]
