# Supplementary material for: Distinct DNA-binding surfaces in the ATPase and linker domains of MutLγ determine its substrate specificities and exert separable functions in meiotic recombination and mismatch repair
Source: PLoS Genet. 2017 May 15;13(5):e1006722. doi: 10.1371/journal.pgen.1006722 (PMC5448812; doi:10.1371/journal.pgen.1006722)
Supplement: S1 Table — (DOCX) [file pgen.1006722.s002.docx]

S1 Table: Fluorescent spore assay data for *mlh1* and *mlh3* DNA-binding mutants.

| **Genotype** | | **Strains** | **N** | **Genetic distance *CEN8-ARG4*** | | | | **Genetic distance *ARG4-THR1*** | | | | **MI Nondisjunctions** | | |
| --- | --- | --- | --- | --- | --- | --- | --- | --- | --- | --- | --- | --- | --- | --- |
|  |  |  |  | **cM ± 95% CI** | **% WT ± 95% CI** | **p**  vs. WT | **p**  vs. *mlh1Δ* | **cM ± 95% CI** | **% WT ± 95% CI** | **p**  vs. WT | **p** vs. *mlh1Δ* | **% ± 95% CI** | **p**  vs. WT | **p**  vs. *mlh1Δ* |
| WT |  | SKY3576x3579 | 1268 | 11.63 ± 0.63 | 100.0 ± 5.42 |  |  | 4.2 ± 0.39 | 100.0 ± 9.2 |  |  | 0.08 ± 0.16 |  |  |
| *mlh1Δ* |  | SKY5083x5084 | 1476 | 7.22 ± 0.53 | 62.0 ± 4.56 | 3×10^-9^ |  | 2.8 ± 0.30 | 66.6 ± 10.7 | 0.015 |  | 1.15 ± 0.54 | 4×10^-4^ |  |
| *MLH1:hphMX4* |  | SKY5087x5088 | 666 | 10.21 ± 0.78 | 87.8 ± 6.71 | 0.32 |  | 4.6 ± 0.56 | 108.5 ± 12.2 | 0.87 |  | 0.15 ± 0.29 | 1 |  |
| *mlh1-R214E* |  | SKY5101x5102 | 679 | 9.50 ± 0.85 | 81.7 ± 7.31 | 0.048 |  | 3.7 ± 0.50 | 87.3 ± 13.6 | 0.71 |  | 0.00 ± 0.0 | 1 |  |
| *mlh1-K253E/K254E* |  | SKY5103x5104 | 687 | 4.73 ± 0.79 | 40.7 ± 6.79 | 7×10^-18^ | 2×10^-4^ | 2.8 ± 0.44 | 65.5 ± 15.9 | 0.057 | 1 | 2.33 ± 1.13 | <1×10^-4^ | 0.057 |
| *mlh1-R273E/R274E* |  | SKY5105x5106 | 697 | 4.95 ± 0.57 | 42.6 ± 4.90 | 1×10^-12^ | 0.02 | 1.9 ± 0.36 | 44.2 ± 19.3 | 2×10^-4^ | 0.1 | 2.87 ± 1.24 | <1×10^-4^ | 6×10^-3^ |
| *mlh1-K286E/R289E* |  | SKY5107x5108 | 687 | 5.82 ± 0.73 | 50.1 ± 6.28 | 1×10^-10^ | 0.17 | 2.8 ± 0.44 | 65.5 ± 15.9 | 0.057 | 1 | 3.20 ± 1.32 | <1×10^-4^ | 1.5×10^-3^ |
| *mlh1-R341E/K344E* |  | SKY5109x5110 | 675 | 6.44 ± 0.76 | 55.4 ± 6.53 | 2×10^-8^ | 0.58 | 2.2 ± 0.40 | 52.7 ± 18.0 | 3×10^-3^ | 0.51 | 2.22 ± 1.11 | <1×10^-4^ | 0.082 |
| *mlh1-R367E/R369E/K370E/R373E* |  | SKY5111x5112 | 682 | 11.14 ± 0.89 | 95.8 ± 7.65 | 0.7 |  | 3.5 ± 0.49 | 83.4 ± 13.9 | 0.55 |  | 0.29 ± 0.4 | 0.28 |  |
| *mlh1-K393E/R394E* |  | SKY5113x5114 | 679 | 10.24 ± 0.77 | 88.0 ± 6.62 | 0.32 |  | 3.5 ± 0.49 | 83.8 ± 13.9 | 0.56 |  | 0.44 ± 0.5 | 0.13 |  |
| *mlh1-K398E/R401E* |  | SKY5115x5116 | 676 | 11.91 ± 0.82 | 102.4 ± 7.05 | 0.58 |  | 3.3 ± 0.48 | 78.9 ± 14.4 | 0.37 |  | 0.00 ± 0 | 1 |  |
|  | |  |  |  |  |  |  |  |  |  |  |  |  |  |
| WT |  | SKY3576x3579 | 1268 | 11.63 ± 0.63 | 100.0 ± 5.42 |  |  | 4.2 ± 0.39 | 100.0 ± 9.2 |  |  | 0.08 ± 0.16 |  |  |
| *mlh3Δ* |  | SKY5085x5086 | 990 | 7.12 ± 0.62 | 61.2 ± 5.33 | 2×10^-7^ |  | 2.6 ± 0.35 | 62.2 ± 13.3 | 0.012 |  | 2.73 ± 1.02 | <1×10^-4^ |  |
| *MLH3:hphMX4* |  | SKY5089x5090 | 685 | 11.82 ± 0.81 | 101.7 ± 6.96 | 0.59 |  | 4.3 ± 0.54 | 102.1 ± 12.5 | 0.99 |  | 0.15 ± 0.29 | 1 |  |
| *mlh3-R171E/R172E/R173E* |  | SKY5121x5122 | 682 | 7.62 ± 0.69 | 65.5 ± 5.93 | 2×10^-4^ |  | 3.6 ± 0.49 | 85.1 ± 13.6 | 0.62 |  | 1.17 ± 0.81 | 1.4×10^-3^ |  |
| *mlh3-R220E/K222E* |  | SKY5123x5124 | 681 | 9.10 ± 0.74 | 78.3 ± 6.36 | 0.037 |  | 4.1 ± 0.53 | 97.4 ± 12.9 | 0.99 |  | 0.29 ± 0.4 | 0.28 |  |
| *mlh3-R316E/R320E/R323E* |  | SKY5125x5126 | 685 | 5.62 ± 0.72 | 48.3 ± 6.19 | 2×10^-11^ |  | 3.1 ± 0.46 | 72.7 ± 15.0 | 0.18 |  | 3.21 ± 1.32 | <1×10^-4^ |  |
| *mlh3-K347E/K351E* |  | SKY5127x5128 | 684 | 8.04 ± 0.81 | 69.1 ± 6.96 | 2×10^-4^ |  | 3.4 ± 0.48 | 79.7 ± 14.3 | 0.40 |  | 0.88 ± 0.7 | 9×10^-3^ |  |
| *mlh3-R401E/K406E/R407E* |  | SKY5129x5130 | 679 | 6.85 ± 0.77 | 58.9 ± 6.62 | 3×10^-7^ |  | 2.5 ± 0.42 | 59.3 ± 16.8 | 0.017 |  | 1.18 ± 0.81 | 1.3×10^-3^ |  |
| *mlh3-K414E/K416E* |  | SKY5131x5132 | 688 | 6.40 ± 0.64 | 55.0 ± 5.50 | 2×10^-7^ |  | 1.7 ± 0.34 | 39.6 ± 20.3 | 3×10^-5^ |  | 1.74 ± 0.98 | <1×10^-4^ |  |
| *mlh3-R419E/K426E* |  | SKY5133x5134 | 678 | 11.28 ± 0.80 | 97.0 ± 6.88 | 0.65 |  | 4.2 ± 0.53 | 99.6 ± 12.6 | 1 |  | 0.15 ± 0.29 | 1 |  |
| *mlh3-K443E/K445E/R448E* |  | SKY5135x5136 | 694 | 6.99 ± 0.66 | 60.1 ± 5.67 | 6×10^-6^ |  | 2.2 ± 0.39 | 52.9 ± 17.5 | 3×10^-3^ |  | 0.58 ± 0.56 | 0.056 |  |
|  | | | | | | | | | | | | | | |
| Standard error calculation for genetic intervals was done using the Stahl lab online tool: http://molbio.uoregon.edu/~fstahl/compare2.php. | | | | | | | | | | | | | | |
| p value for genetic distance were calculated by G test. | | | | | | | | | | | | | | |
| p value for MI nondisjunction were calculated by Fisher's exact test (two-tailed p value). | | | | | | | | | | | | | | |
