# Supplementary material for: Distinct DNA-binding surfaces in the ATPase and linker domains of MutLγ determine its substrate specificities and exert separable functions in meiotic recombination and mismatch repair
Source: PLoS Genet. 2017 May 15;13(5):e1006722. doi: 10.1371/journal.pgen.1006722 (PMC5448812; doi:10.1371/journal.pgen.1006722)
Supplement: S2 Table — (DOCX) [file pgen.1006722.s003.docx]

**S2 Table: MMR data for *mlh1* and *mlh3* DNA-binding mutants and tagged strains.**

| **Lys+ reversion** | | **Strain** | **N** | **Frequency ×10^-3^ (Mean ± SD)** | **Frequency (normalized to *mlh1Δ*) (Mean ± SD)** | **p** |
| --- | --- | --- | --- | --- | --- | --- |
| WT |  | SKY5139 | 3 | 0.1 ± 0.0 | 0.00 ± 0.001 |  |
| *mlh1Δ* |  | SKY5175 | 6 | 43.8 ± 14.1 | 1.00 ± 0.323 | 0.0013 |
| *MLH1:hphMX4* |  | SKY5088 | 3 | 0.1 ± 0.0 | 0.00 ± 0.001 | 0.8957 |
| *mlh1-R214E* |  | SKY5089 | 4 | 0.5 ± 0.2 | 0.01 ± 0.004 | 0.0126 |
| *mlh1-K253E/K254E* |  | SKY5090 | 5 | 36.0 ± 15.5 | 0.82 ± 0.354 | 0.0081 |
| *mlh1-R273E/R274E* |  | SKY5091 | 6 | 36.0 ± 9.5 | 0.82 ± 0.217 | 0.0004 |
| *mlh1-K286E/R289E* |  | SKY5092 | 6 | 29.4 ± 12.0 | 0.67 ± 0.275 | 0.0047 |
| *mlh1-R341E/K344E* |  | SKY5093 | 3 | 31.0 ± 1.9 | 0.71 ± 0.045 | 0.0001 |
| *mlh1-R367E/R369E/K370E/R373E* |  | SKY5094 | 5 | 0.6 ± 0.1 | 0.01 ± 0.002 | 0.0002 |
| *mlh1-K393E/R394E* |  | SKY5095 | 6 | 21.1 ± 9.9 | 0.48 ± 0.226 | 0.0092 |
| *mlh1-K398E/R401E* |  | SKY5096 | 6 | 32.0 ± 17.2 | 0.73 ± 0.392 | 0.0172 |
|  | | | | | | |
| **Lys+ reversion** | | **Strain** | **N** | **Frequency ×10^-6^ (Mean ± SD)** | **Frequency (normalized to *mlh3Δ*) (Mean ± SD)** | **p** |
| WT |  | SKY5139 | 6 | 33 ± 3 | 0.19 ± 0.045 |  |
| *mlh3Δ* |  | SKY5176 | 6 | 172 ± 22 | 1.00 ± 0.320 | <0.0001 |
| *MLH3:hphMX4* |  | SKY5199 | 6 | 42 ± 5 | 0.24 ± 0.068 | 0.0036 |
| *mlh3-R171E/R172E/R173E* |  | SKY5200 | 6 | 161 ± 22 | 0.94 ± 0.318 | <0.0001 |
| *mlh3-R220E/K222E* |  | SKY5201 | 6 | 151 ± 4 | 0.88 ± 0.063 | <0.0001 |
| *mlh3-R316E/R320E/R323E* |  | SKY5202 | 6 | 182 ± 15 | 1.06 ± 0.215 | <0.0001 |
| *mlh3-K347E/K351E* |  | SKY5203 | 5 | 131 ± 9 | 0.76 ± 0.113 | <0.0001 |
| *mlh3-R401E/K406E/R407E* |  | SKY5204 | 6 | 98 ± 4 | 0.57 ± 0.064 | <0.0001 |
| *mlh3-K414E/K416E* |  | SKY5205 | 6 | 89 ± 4 | 0.52 ± 0.062 | <0.0001 |
| *mlh3-R419E/K426E* |  | SKY5206 | 6 | 89 ± 6 | 0.52 ± 0.088 | <0.0001 |
| *mlh3-K443E/K445E/R448E* |  | SKY5207 | 6 | 61 ± 7 | 0.36 ± 0.104 | <0.0001 |
|  | | | | | | |
| **Thr+ reversion** | | **Strain** | **N** | **Frequency ×10^-6^ (Mean ± SD)** | **Frequency (normalized to *mlh1Δ*) (Mean ± SD)** | **p** |
| WT |  | SKY5137 | 3 | 0 ± 0 | 0.00 ± 0.000 |  |
| *mlh1Δ* |  | SKY5173 | 3 | 102 ± 7 | 1.00 ± 0.067 | <0.0001 |
| *MLH1:hphMX4* |  | SKY5177 | 3 | 0 ± 0 | 0.00 ± 0.000 |  |
| *mlh1-R214E* |  | SKY5178 | 3 | 1 ± 1 | 0.01 ± 0.006 | 0.1583 |
| *mlh1-K253E/K254E* |  | SKY5179 | 3 | 53 ± 9 | 0.52 ± 0.090 | 0.0005 |
| *mlh1-R273E/R274E* |  | SKY5180 | 3 | 88 ± 18 | 0.86 ± 0.173 | 0.0011 |
| *mlh1-K286E/R289E* |  | SKY5181 | 3 | 46 ± 6 | 0.46 ± 0.063 | 0.0002 |
| *mlh1-R341E/K344E* |  | SKY5182 | 3 | 65 ± 12 | 0.64 ± 0.114 | 0.0007 |
| *mlh1-R367E/R369E/K370E/R373E* |  | SKY5183 | 3 | 0 ± 0 | 0.00 ± 0.003 |  |
| *mlh1-K393E/R394E* |  | SKY5184 | 3 | 35 ± 2 | 0.35 ± 0.022 | <0.0001 |
| *mlh1-K398E/R401E* |  | SKY5185 | 3 | 76 ± 3 | 0.74 ± 0.029 | <0.0001 |
|  | | | | | | |
| **Lys+ reversion – tagged strains** | | **Strain** | **N** | **Frequency ×10^-6^ (Mean ± SD)** | **Frequency (normalized to WT) (Mean ± SD)** | **p** |
| *HisFlag-MLH1* | | SKY5400 | 3 | 48 ± 9 | 1.37 ± 0.25 | 0.006 |
| *HisFlag-MLH3* | | SKY5401 | 3 | 34 ± 9 | 0.97 ± 0.25 | 0.8023 |
|  | | | | | | |
| N is the number of independent cultures.  p values were calculated by unpaired t test. | | | | | | |
