# Supplementary material for: Distinct DNA-binding surfaces in the ATPase and linker domains of MutLγ determine its substrate specificities and exert separable functions in meiotic recombination and mismatch repair
Source: PLoS Genet. 2017 May 15;13(5):e1006722. doi: 10.1371/journal.pgen.1006722 (PMC5448812; doi:10.1371/journal.pgen.1006722)
Supplement: S3 Table — (DOCX) [file pgen.1006722.s004.docx]

**S3 Table: Fluorescent spore assay data of *mlh1* and *mlh3* mutants in *mms4Δ* and *msh5Δ* backgrounds and tagged strains.**

| **Genotype** | **Strains** | **N** | **Genetic distance *CEN8-ARG4*** | | **MI Nondisjunctions**  **% ± 95% CI** |
| --- | --- | --- | --- | --- | --- |
|  |  |  | **cM ± 95% CI** | **% WT ± 95% CI** |  |
| WT | SKY3576x3579 | 1268 | 11.63 ± 0.63 | 100.0 ± 5.4 | 0.08 ± 0.16 |
| *mms4Δ* | SKY5531x5532 | 689 | 11.47 ± 0.89 | 98.6 ± 7.7 | 0.87 ± 0.69 |
| *mlh1Δ* | SKY5083x5084 | 1476 | 7.22 ± 0.53 | 62.0 ± 4.6 | 1.15 ± 0.54 |
| *mlh1Δ* *mms4Δ* | SKY5558x5559 | 595 | 3.19 ± 0.50 | 27.5 ± 4.3 | 3.03 ± 1.38 |
| *mlh3Δ* | SKY5085x5086 | 990 | 7.12 ± 0.62 | 61.2 ± 5.3 | 2.73 ± 1.02 |
| *mlh3Δ* *mms4Δ* | SKY5564x5565 | 601 | 2.50 ± 0.64 | 21.5 ± 5.5 | 3.00 ± 1.36 |
| *mlh1-Nterm* | SKY5107x5108 | 687 | 5.82 ± 0.73 | 50.1 ± 6.3 | 3.20 ± 1.32 |
| *mlh1-Nterm mms4Δ* | SKY5560x5561 | 522 | 1.92 ± 0.42 | 16.5 ± 3.6 | 4.02 ± 1.69 |
| *mlh3-Nterm* | SKY5125x5126 | 685 | 5.62 ± 0.72 | 48.3 ± 6.2 | 3.21 ± 1.32 |
| *mlh3-Nterm mms4Δ* | SKY5566x5567 | 601 | 1.25 ± 0.32 | 10.7 ± 2.8 | 3.33 ± 1.43 |
| *mlh1-linker* | SKY5113x5114 | 679 | 10.24 ± 0.77 | 88.0 ± 6.6 | 0.44 ± 0.5 |
| *mlh1-linker mms4Δ* | SKY5562x5563 | 311 | 7.07 ± 0.99 | 60.8 ± 8.5 | 2.89 ± 1.86 |
| *mlh3-linker* | SKY5131x5132 | 688 | 6.40 ± 0.64 | 55.0 ± 5.5 | 1.74 ± 0.98 |
| *mlh3-linker mms4Δ* | SKY5568x5569 | 600 | 1.33 ± 0.33 | 11.5 ± 2.8 | 2.83 ± 1.33 |
|  |  |  |  |  |  |
| WT | SKY3576x3579 | 1268 | 11.63 ± 0.63 | 100.0 ± 5.4 | 0.08 ± 0.16 |
| *msh5Δ* | SKY3580x3581 | 685 | 3.50 ± 0.75 | 30.1 ± 6.4 | 13.72 ± 2.58 |
| *mlh1Δ* | SKY5083x5084 | 1476 | 7.22 ± 0.53 | 62.0 ± .6 | 1.15 ± 0.54 |
| *mlh1Δ* *msh5Δ* | SKY5570x5571 | 600 | 4.50 ± 0.58 | 38.7 ± 5.0 | 8.67 ± 2.25 |
| *mlh3Δ* | SKY5085x5086 | 990 | 7.12 ± 0.62 | 61.2 ± 5.3 | 2.73 ± 1.02 |
| *mlh3Δ* *msh5Δ* | SKY5576x5577 | 602 | 3.16 ± 0.81 | 27.1 ± 7.0 | 13.62 ± 2.74 |
| *mlh1-Nterm* | SKY5107x5108 | 687 | 5.82 ± 0.73 | 50.1 ± 6.3 | 3.20 ± 1.32 |
| *mlh1-Nterm msh5Δ* | SKY5572x5573 | 601 | 3.33 ± 0.68 | 28.6 ± 5.8 | 11.81 ± 2.58 |
| *mlh3-Nterm* | SKY5125x5126 | 685 | 5.62 ± 0.72 | 48.3 ± 6.2 | 3.21 ± 1.32 |
| *mlh3-Nterm msh5Δ* | SKY5578x5579 | 593 | 2.61 ± 0.46 | 22.5 ± 4.0 | 11.64 ± 2.58 |
| *mlh1-linker* | SKY5113x5114 | 679 | 10.24 ± 0.77 | 88.0 ± 6.6 | 0.44 ± 0.5 |
| *mlh1-linker msh5Δ* | SKY5574x5575 | 600 | 2.92 ± 0.48 | 25.1 ± 4.1 | 9.83 ± 2.38 |
| *mlh3-linker* | SKY5131x5132 | 688 | 6.40 ± 0.64 | 55.0 ± 5.5 | 1.74 ± 0.98 |
| *mlh3-linker msh5Δ* | SKY5580x5581 | 600 | 2.00 ± 0.40 | 17.2 ± 3.4 | 13.67 ± 2.75 |
|  |  |  |  |  |  |
| *HisFlag-Mlh1* | SKY5386x5387 | 700 | 13.21 ± 0.79 | 117 ± 7.0  ±± | 0.57 ± 0.56 |
| *HisFlag-Mlh3* | SKY5388x5389 | 702 | 9.12 ± 0.67 | 81 ± 6.0 | 0.28 ± 0.39 |
|  | | | | | |
| N is the number of tetrads scored. | | | | | |
