# Supplementary material for: Distinct DNA-binding surfaces in the ATPase and linker domains of MutLγ determine its substrate specificities and exert separable functions in meiotic recombination and mismatch repair
Source: PLoS Genet. 2017 May 15;13(5):e1006722. doi: 10.1371/journal.pgen.1006722 (PMC5448812; doi:10.1371/journal.pgen.1006722)
Supplement: S4 Table — (DOCX) [file pgen.1006722.s005.docx]

**S4 Table: List of yeast strains.**

| **Strain** | **MAT** | **Genotype** | **Source/Ref.** |
| --- | --- | --- | --- |
| SKY3576 | a | *ho::LYS2, lys2, ura3, leu2::hisG, trp1::hisG, THR1::Cerulean-TRP1* | Thacker *et al.* 2011 |
| SKY3579 | α | *ho::LYS2, lys2, ura3, leu2::hisG, trp1::hisG, CEN8::tdTomato-LEU2, ARG4::Yellow-URA3* | Thacker *et al.* 2011 |
| SKY3580 | a | *ho::LYS2, lys2, ura3, leu2::hisG, trp1::hisG, THR1::Cerulean-TRP1, msh5Δ::kanMX4* |  |
| SKY3581 | α | *ho::LYS2, lys2, ura3, leu2::hisG, trp1::hisG, CEN8::tdTomato-LEU2, ARG4::Yellow-URA3, msh5Δ::kanMX4* |  |
| SKY5083 | a | *ho::LYS2, lys2, ura3, leu2::hisG, trp1::hisG, THR1::Cerulean-TRP1, mlh1Δ::kanMX4* |  |
| SKY5084 | α | *ho::LYS2, lys2, ura3, leu2::hisG, trp1::hisG, CEN8::tdTomato-LEU2, ARG4::Yellow-URA3, mlh1Δ::kanMX4* |  |
| SKY5085 | a | *ho::LYS2, lys2, ura3, leu2::hisG, trp1::hisG, THR1::Cerulean-TRP1, mlh3Δ::kanMX4* |  |
| SKY5086 | α | *ho::LYS2, lys2, ura3, leu2::hisG, trp1::hisG, CEN8::tdTomato-LEU2, ARG4::Yellow-URA3, mlh3Δ::kanMX4* |  |
| SKY5087 | a | *ho::LYS2, lys2, ura3, leu2::hisG, trp1::hisG, THR1::Cerulean-TRP1, MLH1::hphMX4* |  |
| SKY5088 | α | *ho::LYS2, lys2, ura3, leu2::hisG, trp1::hisG, CEN8::tdTomato-LEU2, ARG4::Yellow-URA3, MLH1::hphMX4* |  |
| SKY5089 | a | *ho::LYS2, lys2, ura3, leu2::hisG, trp1::hisG, THR1::Cerulean-TRP1, MLH3::hphMX4* |  |
| SKY5090 | α | *ho::LYS2, lys2, ura3, leu2::hisG, trp1::hisG, CEN8::tdTomato-LEU2, ARG4::Yellow-URA3, MLH3::hphMX4* |  |
| SKY5101 | a | *ho::LYS2, lys2, ura3, leu2::hisG, trp1::hisG, THR1::Cerulean-TRP1, mlh1-R214::hphMX4* |  |
| SKY5102 | α | *ho::LYS2, lys2, ura3, leu2::hisG, trp1::hisG, CEN8::tdTomato-LEU2, ARG4::Yellow-URA3, mlh1-R214::hphMX4* |  |
| SKY5103 | a | *ho::LYS2, lys2, ura3, leu2::hisG, trp1::hisG, THR1::Cerulean-TRP1, mlh1-K253E/K254E::hphMX4* |  |
| SKY5104 | α | *ho::LYS2, lys2, ura3, leu2::hisG, trp1::hisG, CEN8::tdTomato-LEU2, ARG4::Yellow-URA3, mlh1-K253E/K254E::hphMX4* |  |
| SKY5105 | a | *ho::LYS2, lys2, ura3, leu2::hisG, trp1::hisG, THR1::Cerulean-TRP1, mlh1-R273E/R274E::hphMX4* |  |
| SKY5106 | α | *ho::LYS2, lys2, ura3, leu2::hisG, trp1::hisG, CEN8::tdTomato-LEU2, ARG4::Yellow-URA3, mlh1-R273E/R274E::hphMX4* |  |
| SKY5107 | a | *ho::LYS2, lys2, ura3, leu2::hisG, trp1::hisG, THR1::Cerulean-TRP1, mlh1-K286E/R289E::hphMX4* |  |
| SKY5108 | α | *ho::LYS2, lys2, ura3, leu2::hisG, trp1::hisG, CEN8::tdTomato-LEU2, ARG4::Yellow-URA3, mlh1-K286E/R289E::hphMX4* |  |
| SKY5109 | a | *ho::LYS2, lys2, ura3, leu2::hisG, trp1::hisG, THR1::Cerulean-TRP1, mlh1-R341E/K344E::hphMX4* |  |
| SKY5110 | α | *ho::LYS2, lys2, ura3, leu2::hisG, trp1::hisG, CEN8::tdTomato-LEU2, ARG4::Yellow-URA3, mlh1-R341E/K344E::hphMX4* |  |
| SKY5111 | a | *ho::LYS2, lys2, ura3, leu2::hisG, trp1::hisG, THR1::Cerulean-TRP1, mlh1-R367E/R369E/K370E/R373E::hphMX4* |  |
| SKY5112 | α | *ho::LYS2, lys2, ura3, leu2::hisG, trp1::hisG, CEN8::tdTomato-LEU2, ARG4::Yellow-URA3, mlh1-R367E/R369E/K370E/R373E::hphMX4* |  |
| SKY5113 | a | *ho::LYS2, lys2, ura3, leu2::hisG, trp1::hisG, THR1::Cerulean-TRP1, mlh1-K393E/R394E::hphMX4* |  |
| SKY5114 | α | *ho::LYS2, lys2, ura3, leu2::hisG, trp1::hisG, CEN8::tdTomato-LEU2, ARG4::Yellow-URA3, mlh1-K393E/R394E::hphMX4* |  |
| SKY5115 | a | *ho::LYS2, lys2, ura3, leu2::hisG, trp1::hisG, THR1::Cerulean-TRP1, mlh1-K398E/R401E::hphMX4* |  |
| SKY5116 | α | *ho::LYS2, lys2, ura3, leu2::hisG, trp1::hisG, CEN8::tdTomato-LEU2, ARG4::Yellow-URA3, mlh1-K398E/R401E::hphMX4* |  |
| SKY5121 | a | *ho::LYS2, lys2, ura3, leu2::hisG, trp1::hisG, THR1::Cerulean-TRP1, mlh3-R171E/R172E/R173E::hphMX4* |  |
| SKY5122 | α | *ho::LYS2, lys2, ura3, leu2::hisG, trp1::hisG, CEN8::tdTomato-LEU2, ARG4::Yellow-URA3, mlh3-R171E/R172E/R173E::hphMX4* |  |
| SKY5123 | a | *ho::LYS2, lys2, ura3, leu2::hisG, trp1::hisG, THR1::Cerulean-TRP1, mlh3-R220E/K222E::hphMX4* |  |
| SKY5124 | α | *ho::LYS2, lys2, ura3, leu2::hisG, trp1::hisG, CEN8::tdTomato-LEU2, ARG4::Yellow-URA3, mlh3-R220E/K222E::hphMX4* |  |
| SKY5125 | a | *ho::LYS2, lys2, ura3, leu2::hisG, trp1::hisG, THR1::Cerulean-TRP1, mlh3-R316E/R320E/R323E::hphMX4* |  |
| SKY5126 | α | *ho::LYS2, lys2, ura3, leu2::hisG, trp1::hisG, CEN8::tdTomato-LEU2, ARG4::Yellow-URA3, mlh3-R316E/R320E/R323E::hphMX4* |  |
| SKY5127 | a | *ho::LYS2, lys2, ura3, leu2::hisG, trp1::hisG, THR1::Cerulean-TRP1, mlh3-K347E/K351E::hphMX4* |  |
| SKY5128 | α | *ho::LYS2, lys2, ura3, leu2::hisG, trp1::hisG, CEN8::tdTomato-LEU2, ARG4::Yellow-URA3, mlh3-K347E/K351E::hphMX4* |  |
| SKY5129 | a | *ho::LYS2, lys2, ura3, leu2::hisG, trp1::hisG, THR1::Cerulean-TRP1, mlh3-R401E/K406E/R407E::hphMX4* |  |
| SKY5130 | α | *ho::LYS2, lys2, ura3, leu2::hisG, trp1::hisG, CEN8::tdTomato-LEU2, ARG4::Yellow-URA3, mlh3-R401E/K406E/R407E::hphMX4* |  |
| SKY5131 | a | *ho::LYS2, lys2, ura3, leu2::hisG, trp1::hisG, THR1::Cerulean-TRP1, mlh3-K414E/K416E::hphMX4* |  |
| SKY5132 | α | *ho::LYS2, lys2, ura3, leu2::hisG, trp1::hisG, CEN8::tdTomato-LEU2, ARG4::Yellow-URA3, mlh3-K414E/K416E::hphMX4* |  |
| SKY5133 | a | *ho::LYS2, lys2, ura3, leu2::hisG, trp1::hisG, THR1::Cerulean-TRP1, mlh3-R419E/K426E::hphMX4* |  |
| SKY5134 | α | *ho::LYS2, lys2, ura3, leu2::hisG, trp1::hisG, CEN8::tdTomato-LEU2, ARG4::Yellow-URA3, mlh3-R419E/K426E::hphMX4* |  |
| SKY5135 | a | *ho::LYS2, lys2, ura3, leu2::hisG, trp1::hisG, THR1::Cerulean-TRP1, mlh3-K443E/K445E/R448E::hphMX4* |  |
| SKY5136 | α | *ho::LYS2, lys2, ura3, leu2::hisG, trp1::hisG, CEN8::tdTomato-LEU2, ARG4::Yellow-URA3, mlh3-K443E/K445E/R448E::hphMX4* |  |
| SKY5137 | α | *ho::LYS2, lys2, leu2::hisG, CAN1, ura3, hom3-10, trp2* | E. Alani (HTY1213) |
| SKY5139 | a | *ho::hisG, ura3, leu2::hisG, ade2::LK, his4xB, lys214::insE-A14* | E. Alani (EAY1062) |
| SKY5173 | α | *ho::LYS2, lys2, leu2::hisG, CAN1, ura3, hom3-10, trp2, mlh1Δ::kanMX4* |  |
| SKY5175 | a | *ho::hisG, ura3, leu2::hisG, ade2::LK, his4xB, lys214::insE-A14, mlh1Δ::kanMX4* |  |
| SKY5176 | a | *ho::hisG, ura3, leu2::hisG, ade2::LK, his4xB, lys214::insE-A14, mlh3Δ::kanMX4* |  |
| SKY5177 | α | *ho::LYS2, lys2, leu2::hisG, CAN1, ura3, hom3-10, trp2, MLH1::hphMX4* |  |
| SKY5178 | α | *ho::LYS2, lys2, leu2::hisG, CAN1, ura3, hom3-10, trp2, mlh1-R214::hphMX4* |  |
| SKY5179 | α | *ho::LYS2, lys2, leu2::hisG, CAN1, ura3, hom3-10, trp2, mlh1-K253E/K254E::hphMX4* |  |
| SKY5180 | α | *ho::LYS2, lys2, leu2::hisG, CAN1, ura3, hom3-10, trp2, mlh1-R273E/R274E::hphMX4* |  |
| SKY5181 | α | *ho::LYS2, lys2, leu2::hisG, CAN1, ura3, hom3-10, trp2, mlh1-K286E/R289E::hphMX4* |  |
| SKY5182 | α | *ho::LYS2, lys2, leu2::hisG, CAN1, ura3, hom3-10, trp2, mlh1-R341E/K344E::hphMX4* |  |
| SKY5183 | α | *ho::LYS2, lys2, leu2::hisG, CAN1, ura3, hom3-10, trp2, mlh1-R367E/R369E/K370E/R373E::hphMX4* |  |
| SKY5184 | α | *ho::LYS2, lys2, leu2::hisG, CAN1, ura3, hom3-10, trp2, mlh1-K393E/R394E::hphMX4* |  |
| SKY5185 | α | *ho::LYS2, lys2, leu2::hisG, CAN1, ura3, hom3-10, trp2, mlh1-K398E/R401E::hphMX4* |  |
| SKY5188 | a | *ho::hisG, ura3, leu2::hisG, ade2::LK, his4xB, lys214::insE-A14, MLH1::hphMX4* |  |
| SKY5189 | a | *ho::hisG, ura3, leu2::hisG, ade2::LK, his4xB, lys214::insE-A14, mlh1-R214E::hphMX4* |  |
| SKY5190 | a | *ho::hisG, ura3, leu2::hisG, ade2::LK, his4xB, lys214::insE-A14, mlh1-K253E/K254E::hphMX4* |  |
| SKY5191 | a | *ho::hisG, ura3, leu2::hisG, ade2::LK, his4xB, lys214::insE-A14, mlh1-R273E/R274E::hphMX4* |  |
| SKY5192 | a | *ho::hisG, ura3, leu2::hisG, ade2::LK, his4xB, lys214::insE-A14, mlh1-K286E/R289E::hphMX4* |  |
| SKY5193 | a | *ho::hisG, ura3, leu2::hisG, ade2::LK, his4xB, lys214::insE-A14, mlh1-R341E/K344E::hphMX4* |  |
| SKY5194 | a | *ho::hisG, ura3, leu2::hisG, ade2::LK, his4xB, lys214::insE-A14, mlh1-R367E/R369E/K370E/R373E::hphMX4* |  |
| SKY5195 | a | *ho::hisG, ura3, leu2::hisG, ade2::LK, his4xB, lys214::insE-A14, mlh1-K393E/R394E::hphMX4* |  |
| SKY5196 | a | *ho::hisG, ura3, leu2::hisG, ade2::LK, his4xB, lys214::insE-A14, mlh1-K398E/R401E::hphMX4* |  |
| SKY5199 | a | *ho::hisG, ura3, leu2::hisG, ade2::LK, his4xB, lys214::insE-A14, MLH3::hphMX4* |  |
| SKY5200 | a | *ho::hisG, ura3, leu2::hisG, ade2::LK, his4xB, lys214::insE-A14, mlh3-R171E/R172E/R173E::hphMX4* |  |
| SKY5201 | a | *ho::hisG, ura3, leu2::hisG, ade2::LK, his4xB, lys214::insE-A14, mlh3-R220E/K222E::hphMX4* |  |
| SKY5202 | a | *ho::hisG, ura3, leu2::hisG, ade2::LK, his4xB, lys214::insE-A14, mlh3-R316E/R320E/R323E::hphMX4* |  |
| SKY5203 | a | *ho::hisG, ura3, leu2::hisG, ade2::LK, his4xB, lys214::insE-A14, mlh3-K347E/K351E::hphMX4* |  |
| SKY5204 | a | *ho::hisG, ura3, leu2::hisG, ade2::LK, his4xB, lys214::insE-A14, mlh3-R401E/K406E/R407E::hphMX4* |  |
| SKY5205 | a | *ho::hisG, ura3, leu2::hisG, ade2::LK, his4xB, lys214::insE-A14, mlh3-K414E/K416E::hphMX4* |  |
| SKY5206 | a | *ho::hisG, ura3, leu2::hisG, ade2::LK, his4xB, lys214::insE-A14, mlh3-R419E/K426E::hphMX4* |  |
| SKY5207 | a | *ho::hisG, ura3, leu2::hisG, ade2::LK, his4xB, lys214::insE-A14, mlh3-K443E/K445E/R448E::hphMX4* |  |
| SKY5386 | a | *ho::LYS2, lys2, ura3, leu2::hisG, trp1::hisG, THR1::Cerulean-TRP1, HisFlag-MLH1::HphMX4* |  |
| SKY5387 | α | *ho::LYS2, lys2, ura3, leu2::hisG, trp1::hisG, CEN8::tdTomato-LEU2, ARG4::Yellow-URA3, HisFlag-MLH1::HphMX4* |  |
| SKY5388 | a | *ho::LYS2, lys2, ura3, leu2::hisG, trp1::hisG, THR1::Cerulean-TRP1, HisFlag-MLH3::HphMX4* |  |
| SKY5389 | α | *ho::LYS2, lys2, ura3, leu2::hisG, trp1::hisG, CEN8::tdTomato-LEU2, ARG4::Yellow-URA3, HisFlag-MLH3::HphMX4* |  |
| SKY5400 | a | *ho::hisG, ura3, leu2::hisG, ade2::LK, his4xB, lys214::insE-A14, HisFlag-MLH1::HphMX4* |  |
| SKY5401 | a | *ho::hisG, ura3, leu2::hisG, ade2::LK, his4xB, lys214::insE-A14, HisFlag-MLH3::HphMX4* |  |
| SKY5530 | a/α | *ho::LYS2/'', lys2/'', ura3/'', leu2::hisG/'', trp1::hisG/'', THR1::Cerulean-TRP1/THR1, CEN8/CEN8::tdTomato-LEU2, ARG4/ARG4::Yellow-URA3, MMS4/mms4∆::kanMX4* |  |
| SKY5531 | a | *ho::LYS2, lys2, ura3, leu2::hisG, trp1::hisG, THR1::Cerulean-TRP1, mms4∆::kanMX4* |  |
| SKY5532 | α | *ho::LYS2, lys2, ura3, leu2::hisG, trp1::hisG, CEN8::tdTomato-LEU2, ARG4::Yellow-URA3, mms4∆::kanMX4* |  |
| SKY5558 | a | *ho::LYS2, lys2, ura3, leu2::hisG, trp1::hisG, THR1::Cerulean-TRP1, mlh1Δ::kanMX4, mms4∆::kanMX4* |  |
| SKY5559 | α | *ho::LYS2, lys2, ura3, leu2::hisG, trp1::hisG, CEN8::tdTomato-LEU2, ARG4::Yellow-URA3, mlh1Δ::kanMX4, mms4∆::kanMX4* |  |
| SKY5560 | a | *ho::LYS2, lys2, ura3, leu2::hisG, trp1::hisG, THR1::Cerulean-TRP1, mlh1-K286E/R289E::hphMX4, mms4∆::kanMX4* |  |
| SKY5561 | α | *ho::LYS2, lys2, ura3, leu2::hisG, trp1::hisG, CEN8::tdTomato-LEU2, ARG4::Yellow-URA3, mlh1-K286E/R289E::hphMX4, mms4∆::kanMX4* |  |
| SKY5562 | a | *ho::LYS2, lys2, ura3, leu2::hisG, trp1::hisG, THR1::Cerulean-TRP1, mlh1-K393E/R394E::hphMX4, mms4∆::kanMX4* |  |
| SKY5563 | α | *ho::LYS2, lys2, ura3, leu2::hisG, trp1::hisG, CEN8::tdTomato-LEU2, ARG4::Yellow-URA3, mlh1-K393E/R394E::hphMX4, mms4∆::kanMX4* |  |
| SKY5564 | a | *ho::LYS2, lys2, ura3, leu2::hisG, trp1::hisG, THR1::Cerulean-TRP1, mlh3Δ::kanMX4, mms4∆::kanMX4* |  |
| SKY5565 | α | *ho::LYS2, lys2, ura3, leu2::hisG, trp1::hisG, CEN8::tdTomato-LEU2, ARG4::Yellow-URA3, mlh3Δ::kanMX4, mms4∆::kanMX4* |  |
| SKY5566 | α | *ho::LYS2, lys2, ura3, leu2::hisG, trp1::hisG, THR1::Cerulean-TRP1, mlh3-R316E/R320E/R323E::hphMX4, mms4∆::kanMX4* |  |
| SKY5567 | a | *ho::LYS2, lys2, ura3, leu2::hisG, trp1::hisG, CEN8::tdTomato-LEU2, ARG4::Yellow-URA3, mlh3-R316E/R320E/R323E::hphMX4, mms4∆::kanMX4* |  |
| SKY5568 | a | *ho::LYS2, lys2, ura3, leu2::hisG, trp1::hisG, THR1::Cerulean-TRP1, mlh3-K414E/K416E::hphMX4, mms4∆::kanMX4* |  |
| SKY5569 | α | *ho::LYS2, lys2, ura3, leu2::hisG, trp1::hisG, CEN8::tdTomato-LEU2, ARG4::Yellow-URA3, mlh3-K414E/K416E::hphMX4, mms4∆::kanMX4* |  |
| SKY5570 | α | *ho::LYS2, lys2, ura3, leu2::hisG, trp1::hisG, THR1::Cerulean-TRP1, mlh1Δ::kanMX4, msh5Δ::kanMX4* |  |
| SKY5571 | a | *ho::LYS2, lys2, ura3, leu2::hisG, trp1::hisG, CEN8::tdTomato-LEU2, ARG4::Yellow-URA3, mlh1Δ::kanMX4, msh5Δ::kanMX4* |  |
| SKY5572 | α | *ho::LYS2, lys2, ura3, leu2::hisG, trp1::hisG, THR1::Cerulean-TRP1, mlh1-K286E/R289E::hphMX4, msh5Δ::kanMX4* |  |
| SKY5573 | a | *ho::LYS2, lys2, ura3, leu2::hisG, trp1::hisG, CEN8::tdTomato-LEU2, ARG4::Yellow-URA3, mlh1-K286E/R289E::hphMX4, msh5Δ::kanMX4* |  |
| SKY5574 | a | *ho::LYS2, lys2, ura3, leu2::hisG, trp1::hisG, THR1::Cerulean-TRP1, mlh1-K393E/R394E::hphMX4, msh5Δ::kanMX4* |  |
| SKY5575 | α | *ho::LYS2, lys2, ura3, leu2::hisG, trp1::hisG, CEN8::tdTomato-LEU2, ARG4::Yellow-URA3, mlh1-K393E/R394E::hphMX4, msh5Δ::kanMX4* |  |
| SKY5576 | α | *ho::LYS2, lys2, ura3, leu2::hisG, trp1::hisG, THR1::Cerulean-TRP1, mlh3Δ::kanMX4, msh5Δ::kanMX4* |  |
| SKY5577 | a | *ho::LYS2, lys2, ura3, leu2::hisG, trp1::hisG, CEN8::tdTomato-LEU2, ARG4::Yellow-URA3, mlh3Δ::kanMX4, msh5Δ::kanMX4* |  |
| SKY5578 | a | *ho::LYS2, lys2, ura3, leu2::hisG, trp1::hisG, THR1::Cerulean-TRP1, mlh3-R316E/R320E/R323E::hphMX4, msh5Δ::kanMX4* |  |
| SKY5579 | α | *ho::LYS2, lys2, ura3, leu2::hisG, trp1::hisG, CEN8::tdTomato-LEU2, ARG4::Yellow-URA3, mlh3-R316E/R320E/R323E::hphMX4, msh5Δ::kanMX4* |  |
| SKY5580 | a | *ho::LYS2, lys2, ura3, leu2::hisG, trp1::hisG, THR1::Cerulean-TRP1, mlh3-K414E/K416E::hphMX4, msh5Δ::kanMX4* |  |
| SKY5581 | α | *ho::LYS2, lys2, ura3, leu2::hisG, trp1::hisG, CEN8::tdTomato-LEU2, ARG4::Yellow-URA3, mlh3-K414E/K416E::hphMX4, msh5Δ::kanMX4* |  |
