# Supplementary material for: Distinct DNA-binding surfaces in the ATPase and linker domains of MutLγ determine its substrate specificities and exert separable functions in meiotic recombination and mismatch repair
Source: PLoS Genet. 2017 May 15;13(5):e1006722. doi: 10.1371/journal.pgen.1006722 (PMC5448812; doi:10.1371/journal.pgen.1006722)
Supplement: S5 Table — (DOCX) [file pgen.1006722.s006.docx]

**S5 Table: List of plasmids.**

| **Plasmid** | **Description** |
| --- | --- |
| pCCB203 | pFastBac-HTbFlag (2xFlag in BamHI site of pFastBac-HTb, from V. Bermudez) |
| pCCB313 | S288c *MLH1* in pFastBac-HtbFLAG (in BamHI/SaIl) |
| pCCB314 | S288c *MLH3* in pFastBac-HtbFLAG (in BamHI/EcoRI) |
| pCCB317 | S288c *MLH1* in pFastBac1 (in BamHI/SalI) |
| pCCB318 | S288c *MLH3* in pFastBac1 (in BamHI/EcoRI) |
| pCCB379 | *mlh3-D523N* in pCCB318 |
| pCCB419 | SK1 *MLH1* (ORF + promoter & terminator) + *hphMX4* cassette in Topo vector |
| pCCB420 | SK1 *MLH3* (ORF + promoter & terminator) + *hphMX4* cassette in Topo vector |
| pCCB423 | *mlh1-R214E* in pCCB419 |
| pCCB424 | *mlh1-K253E/K254E* in pCCB419 |
| pCCB425 | *mlh1-R273E/R274E* in pCCB419 |
| pCCB426 | *mlh1-K286E/R289E* in pCCB419 |
| pCCB427 | *mlh1-R341E/K344E* in pCCB419 |
| pCCB428 | *mlh1-R367E/R369E*/K370E/R373E in pCCB419 |
| pCCB429 | *mlh1-K393E/R394E* in pCCB419 |
| pCCB430 | *mlh1-K398E/R401E* in pCCB419 |
| pCCB433 | *mlh3-R171E/R172E/R173E* in pCCB420 |
| pCCB434 | *mlh3-R220E/K222E* in pCCB420 |
| pCCB435 | *mlh3-R316E/K320E/R323E* in pCCB420 |
| pCCB436 | *mlh3-K347E/K351E* in pCCB420 |
| pCCB437 | *mlh3-R401E/K406E/R407E* in pCCB420 |
| pCCB438 | *mlh3-K414E/K416E* in pCCB420 |
| pCCB439 | *mlh3-R419E/K426E* in pCCB420 |
| pCCB440 | *mlh3-K443E/K445E/R448E* in pCCB420 |
| pCCB573 | *HisFlag-MLH1* in pCCB419 |
| pCCB574 | *HisFlag-MLH3* in pCCB420 |
| pCCB593 | *mlh1-K286E/R289E* in pCCB313 |
| pCCB594 | *mlh1-K393E/R394E* in pCCB313 |
| pCCB595 | *mlh3-R316E/K320E/R323E* in pCCB318 |
| pCCB596 | *mlh3-K414E/K416E* in pCCB318 |
