# Supplementary material for: Distinct DNA-binding surfaces in the ATPase and linker domains of MutLγ determine its substrate specificities and exert separable functions in meiotic recombination and mismatch repair
Source: PLoS Genet. 2017 May 15;13(5):e1006722. doi: 10.1371/journal.pgen.1006722 (PMC5448812; doi:10.1371/journal.pgen.1006722)
Supplement: S6 Table — (DOCX) [file pgen.1006722.s007.docx]

**S6 Table: List of oligonucleotides.**

| **Oligo** | **Sequence (5'-3')** |
| --- | --- |
| cb095 | CTAGTATAGAGCCGGCGCGCCATGTCTAGATAGCGTTAGGTCTGCCGAATAGTACTACTCGGATCCCGAGCGAACCACGC |
| cb096 | GCGTGGTTCGCTCGGGATCCGAGTAGTACTATTCGGCAGAGGATTCGAATAGGCCTAATCGAATTCCGCCATCGATGCAC |
| cb097 | GTGCATCGATGGCGGAATTCGATTAGGCCTATTCGAATCCAGACGCGAGTAGATCTTCACGGTACCCGCGGTTACCCGTG |
| cb098 | CACGGGTAACCGCGGGTACCGTGAAGATCTACTCGCGTCTCCTAACGCTATCTAGACATGGCGCGCCGGCTCTATACTAG |
| cb099 | TTTTTTTTTTTTTTTTTTTTTTTTTTTTTTTTTTTTTTTTTTTTTTTTTTTTTTTTTTTTTTTTTTTTTTTTTTTTTTTT |
| cb100 | GCGTGGTTCGCTCGGGATCCGAGTAGTACTATTCGGCAGACCTAACGCTATCTAGACATGGCGCGCCGGCTCTATACTAG |
| cb111 | CAggatccACCATGTCTCTCAGAATAAAAGCACT |
| cb112 | CTACgtcgacTTAACACCTCTCAAAAACTT |
| cb113 | CAggatccACCATGAGCCAGCATATTAGGAAA |
| cb114 | CAgaattcTTACTTCAATTCTGCAATGGGTA |
| cb187 | CTATTAGTTCTAGTTAATCAACATGCTTGCG |
| cb188 | CGCAAGCATGTTGATTAACTAGAACTAATAG |
| cb256 | CTAGTATAGAGCCGGCGCGCCATGTCTAGATAGCGTTAGGTCTGCCGAATAGTACTACTCGGATCCCGAGCGAACCACGC-biot |
| cb316 | TTTTTTTTTTTTTTTTTTTTTTTTTTTTTTTTTTTTTTTTTTTTTTTTTTTTTTTTTTTTTTTTTTTTTTTTTTTTTTTT-Biot |
| cb412 | ATAGTGATAGTAAATGGAAGGTAAAAATAACATAGACCTATCAATAAGCAAGATCTGTTTAGCTTGCCTCGTCC |
| cb413 | CTCAGGAAATAAACAAAAAACTTTGGTATTACAGCCAAAACGTTTTAAAGTGGATGGCGGCGTTAGTATCG |
| cb414 | GCGAGGCTTTCAAGGAAGAATGAACGTGAACTCGTCAACTCAAAAAGAAAAGATCTGTTTAGCTTGCCTCGTCC |
| cb415 | GCGCAATTTAAAATGCAGGCGACAAACCTTGTTCCAAGATTAAGGTTCTCTGGATGGCGGCGTTAGTATCG |
| cb416 | CTTTAAAACGTTTTGGCTGTAATACCAAAGTTTTTTGTTTATTTCCTGAGAGATCTGTTTAGCTTGCCTCGTCC |
| cb417 | GATGAATCGTTAAAGGAAAGGCATACACTTTCAAATGAAACACAATCACATGGATGGCGGCGTTAGTATCG |
| cb345 | GCGTGG*TT*CG*CT*CG*GGATCCGAGTAGTACTATTCGGCAGAGGATTCGAATAGGCCTAATCGAATTCCGCCATCGATGCAC |
| cb347 | GCGTGGTTCGCTCGGG*AT*CC*GA*GT*AGTACTATTCGGCAGAGGATTCGAATAGGCCTAATCGAATTCCGCCATCGATGCAC |
| cb349 | GCGTGGTTCGCTCGGGATCCGAGTAG*TA*CT*AT*TC*GGCAGAGGATTCGAATAGGCCTAATCGAATTCCGCCATCGATGCAC |
| cb351 | GCGTGGTTCGCTCGGGATCCGAGTAGTACTATTCGG*CA*GA*GG*AT*TCGAATAGGCCTAATCGAATTCCGCCATCGATGCAC |
| cb361 | TTTTTTTT*TTTTTTTT*TTTTTTTT*TTTTTTTT*TTTTTTTT*TTTTTTTT*TTTTTTTT*TTTTTTTT*TTTTTTTT*TTTTTTTT-Biot |
| cb363 | GTGCAT*CG*AT*GG*CG*GAATTCGATTAGGCCTATTCGAATCCAGACGCGAGTAGATCTTCACGGTACCCGCGGTTACCCGTG |
| cb365 | GTGCATCGATGGCGGA*AT*TC*GA*TT*AGGCCTATTCGAATCCAGACGCGAGTAGATCTTCACGGTACCCGCGGTTACCCGTG |
| cb367 | GTGCATCGATGGCGGAATTCGATTAG*GC*CT*AT*TC*GAATCCAGACGCGAGTAGATCTTCACGGTACCCGCGGTTACCCGTG |
| cb369 | GTGCATCGATGGCGGAATTCGATTAGGCCTATTCGA*AT*CC*AG*AC*GCGAGTAGATCTTCACGGTACCCGCGGTTACCCGTG |
| cb424 | TACCAGTCGGTTGGAAGTGAAGA |
| cb425 | CTCTCATAAAAGGACTCGG |
| cb426 | AGTGCGTCTACCAAGTATGTTAC |
| cb427 | CTAGGGCTACATTTATAATATC |
| cb428 | GAGAACCTTAATCTTGGAACAAGGTTTGTCGCCTGCATTTTAAATTGGAAAGATCTGTTTAGCTTGCCTCGTCC |
| cb429 | ATGAGGTAATCTTCTGTTTTGCATATCCGCGATGCAGGCGACAAACCTTGTGGATGGCGGCGTTAGTATCG |
| cb430 | GTCCAGGATAGGATTAGGACTGTGTTCAATAA |
| cb431 | TTATTGAACACAGTCTCAATCCTATCCTGGAC |
| cb432 | TTGAATTTCATATCCGAAGAGTCCATTTCACCAA |
| cb433 | TTGGTGAAATGGACTCTTCGGATATGAAATTCAA |
| cb434 | GTGACATGTGATCCTCTAGAAGAAGCTTTGAACAGTGTTTA |
| cb435 | TAAACACTGTTCAAAGCTTCTTCTAGAGGATCACATGTCAC |
| cb436 | TCCAATTATCTGCCAGAGGGCAACGAACCTTTTATTTATTTG |
| cb437 | CAAATAAATAAAAGGTTCGTTGCCCTCTGGCAGATAATTGGA |
| cb438 | TGCCATTGATACTTCAGAAACTTTCGAGGCTTCTTCAATCTC |
| cb439 | GAGATTGAAGAAGCCTCGAAAGTTTCTGAAGTATCAATGGCA |
| cb442 | CAACTAAGGAAAGCGGAAGAACAAGAGAATAAACT |
| cb443 | AGTTTATTCTCTTGTTCTTCCGCTTTCCTTAGTTG |
| cb444 | AAAAGACAAGAGAATGAACTAGTCGAAATAGATGCTTCACAA |
| cb445 | TTGTGAAGCATCTATTTCGACTAGTTCATTCTCTTGTCTTTT |
| cb450 | CTCTGAACACTTGTCCAG |
| cb451 | AGATAATGAACTCATGGA |
| cb452 | GTGATGGAGAGGGAGTGT |
| cb453 | GTGGGTACCGTTGTTAGC |
| cb454 | CTGTATAATTTACCTGTCGAGGAAGAAATACTAAAGGAAGAACCC |
| cb455 | GGGTTCTTCCTTTAGTATTTCTTCCTCGACAGGTAAATTATACAG |
| cb456 | ATACAGAAGTTCTTTTCGAATCAGAGAACATAACTGAGGGG |
| cb457 | CCCCTCAGTTATGTTCTCTGATTCGAAAAGAACTTCTGTAT |
| cb458 | GAGTTTACTCAAAACTGAAAGCGTGGGAGAACCGTATGAATCACATCCCGTCTTC |
| cb459 | GAAGACGGGATGTGATTCATACGGTTCTCCCACGCTTTCAGTTTTGAGTAAACTC |
| cb460 | GTTACAAGATCCAGCAGAGAAAATTGTTGAACCTTCCCATATACG |
| cb461 | CGTATATGGGAAGGTTCAACAATTTTCTCTGCTGGATCTTGTAAC |
| cb462 | CAACATTGCCTGATAGTGAAATCCAAATCAGTGAAGAAAATCAAGTATTAAATTC |
| cb463 | GAATTTAATACTTGATTTTCTTCACTGATTTGGATTTCACTATCAGGCAATGTTG |
| cb464 | TCAAGTATTAAATTCAGAAATGGAGATTGCACGCATTAAT |
| cb465 | ATTAATGCGTGCAATCTCCATTTCTGAATTTAATACTTGA |
| cb466 | TCAAAAATGAAGATTGCAGAGATTAATTCATACATAGGAGAACCCGTTGTCAATG |
| cb467 | CATTGACAACGGGTTCTCCTATGTATGAATTAATCTCTGCAATCTTCATTTTTGA |
| cb468 | GTACAATAAACTACGAAGAAATCGAGAATATAGAAATCGACGGTCAGAAAT |
| cb469 | ATTTCTGACCGTCGATTTCTATATTCTCGATTTCTTCGTAGTTTATTGTAC |
| cb470 | GACACCATAGAAAGTGATGAGAATGAGGAGAGTCTCGAACAAGCCCAAGTGGTAGAG |
| cb471 | CTCTACCACTTGGGCTTGTTCGAGACTCTCCTCATTCTCATCACTTTCTATGGTGTC |
| cb696 | GCCATGGGATCGGATTATAAAGATGACGATGACAAGGATTACAAGGACGATGATGACAAAGGTGGATCCACCATGTCTCTCAGAATAAAAG |
| cb697 | GCCCTGAAAATACAGGTTTTCGGTCGTTGGGATATCGTAATCGTGATGGTGATGGTGATGGTAGTACGACATTGCTTATTGATAGGTCTATG |
| cb698 | GCCATGGGATCGGATTATAAAGATGACGATGACAAGGATTACAAGGACGATGATGACAAAGGTGGATCCACCATGAGCCAGCATATTAGG |
| cb699 | GCCCTGAAAATACAGGTTTTCGGTCGTTGGGATATCGTAATCGTGATGGTGATGGTGATGGTAGTACGACATTTTCTTTTTGAGTTGACGAG |
|  | |
| ‘-BIOT’ indicates the position of a 3'-biotin modification.  ‘*’ indicates the position of a phosphorothioate modification. | |
